# Circulating Mitochondrial DNA as Biomarker Linking Environmental Chemical Exposure to Early Preclinical Lesions Elevation of mtDNA in Human Serum after Exposure to Carcinogenic Halo-Alkane-Based Pesticides

**DOI:** 10.1371/journal.pone.0064413

**Published:** 2013-05-31

**Authors:** Lygia T. Budnik, Stefan Kloth, Xaver Baur, Alexandra M. Preisser, Heidi Schwarzenbach

**Affiliations:** 1 Institute for Occupational and Maritime Medicine, Department of Occupational Health, School of Medicine, University of Hamburg, Hamburg, Germany; 2 Institute for Occupational Medicine, Campus Benjamin Franklin, Charité-School of Medicine, Berlin, Germany; 3 Norwegian Center of Maritime Medicine, Haukeland University Hospital, Bergen, Norway; 4 Department of Tumor Biology, School of Medicine, University of Hamburg, Hamburg, Germany; Johns Hopkins University, United States of America

## Abstract

There is a need for a panel of suitable biomarkers for detection of environmental chemical exposure leading to the initiation or progression of degenerative diseases or potentially, to cancer. As the peripheral blood may contain increased levels of circulating cell-free DNA in diseased individuals, we aimed to evaluate this DNA as effect biomarker recognizing vulnerability after exposure to environmental chemicals. We recruited 164 individuals presumably exposed to halo-alkane-based pesticides. Exposure evaluation was based on human biomonitoring analysis; as biomarker of exposure parent halo-methanes, -ethanes and their metabolites, as well as the hemoglobin-adducts methyl valine and hydroxyl ethyl valine in blood were used, complemented by expert evaluation of exposure and clinical intoxication symptoms as well as a questionnaire. Assessment showed exposures to halo alkanes in the concentration range being higher than non-cancer reference doses (RfD) but (mostly) lower than the occupational exposure limits. We quantified circulating DNA in serum from 86 individuals with confirmed exposure to off-gassing halo-alkane pesticides (in storage facilities or in home environment) and 30 non-exposed controls, and found that exposure was significantly associated with elevated serum levels of circulating mitochondrial DNA (in size of 79 bp, mtDNA-79, p = 0.0001). The decreased integrity of mtDNA (mtDNA-230/mtDNA-79) in exposed individuals implicates apoptotic processes (p = 0.015). The relative amounts of mtDNA-79 in serum were positively associated with the lag-time after intoxication to these chemicals (r = 0.99, p<0.0001). Several months of post-exposure the specificity of this biomarker increased from 30% to 97% in patients with intoxication symptoms. Our findings indicate that mitochondrial DNA has a potential to serve as a biomarker recognizing vulnerable risk groups after exposure to toxic/carcinogenic chemicals.

## Introduction

Although epidemiological and toxicological studies provide important evidence for the role of environmental exposure in initiation or progression of degenerative diseases and cancer [1], [2], [3], [4], there is still a challenge to correlate exposure with disease prevalence. In fact, objectively verifiable, individual exposure data (and possible confounder levels) appear to be a major problem allowing to draw a link between the environmental exposure and the cause of disease. In case control studies the collection of exposure data before disease onset (using questionnaires) is retrospective and performed in individuals who are already sick. Except for compounds that accumulate in the body, the use of exposure biomarkers is limited in these studies. It is challenging to date precisely the onset of disease, or to define the periods of vulnerability and the effects of repeated insults, or to assess objectively cumulative exposure. To elaborate environmental risk factors, human precancerous cells removed by biopsy may provide data on basic biology of cancer development. Samples from non-symptomatic, occupationally exposed individuals offer another possibility to correlate exposure with disease risk.

Halogenated hydrocarbons (methyl bromide, ethylene dichloride, ethylene bromide, methyl chloride, dichloromethane or carbon tetra-chloro-methane) are often used in pesticide mixtures for fumigation of export or storage products and wooden pellets [5], [6], [7]. Methyl and ethyl halides are both toxic and potential human carcinogens with some common biochemical effects and metabolic fate [6], including their ability of alkylating macromolecule (hemoglobin/DNA) [8]. These halogenated hydrocarbons quickly metabolize leaving only a short time slot to measure a biomarker of exposure (such as i.e., methyl bromide in blood). Hemoglobin adducts could be regarded as both a long term exposure biomarker and a useful surrogate effect marker for possible formation of DNA adducts [8].

There is a recognized need for a biomarker reflecting early preclinical stages of disease allowing to monitor vulnerable groups. Rappaport and Smith [9] suggested that environmental exposure and disease risk analysis should be based on internal biological marker levels raised upon exposure under consideration of life style factors. However, such analyses have to include toxicological exposure science. Searching for a biomarker representing an early step on the path towards disease risk, so far, surrogate tissues are mainly used. This is based on the hypothesis that the extension of genetic damage in surrogate cells (i.e., peripheral lymphocytes) reflects early carcinogenic events in the target tissues. An increase in chromosomal aberrations as a risk indicator in occupationally and environmentally exposed cohorts has been well documented [10], [11]. To date, the best markers available are the micronucleus test (MNT) and the frequency of chromosomal aberrations reflecting the predisposition for DNA damage in peripheral lymphocytes from exposed individuals [12], [13], [14].

To monitor early preclinical stages of disease, a minimally invasive assay could be eligible. It is well documented that peripheral blood contains nucleic acids, such as DNA and RNA, throughout the body [15], [16], [17], [18], [19]. The detection of increased levels of circulating nucleic acids in plasma or serum of patients with different benign (e.g., inflammatory diseases, diabetes, tissue trauma) and malignant diseases (different histo-pathological cancer entities) as well as altered physiological states (e.g., pregnancy, infections) may provide an easily accessible source of genetic material and insights into the biology of diseases. Circulating DNA is composed of both genomic DNA (gDNA) and mitochondrial DNA (mtDNA). Interestingly, the levels of cell-free mtDNA and gDNA do not correlate in some diseases, indicating the different nature of circulating mtDNA and gDNA, and the different pathophysiologic mechanisms involved in their release and DNA degradation. In contrast to two copies of gDNA, a single cell contains up to several hundred copies of mtDNA. Whereas gDNA usually circulates in a cell-free form, circulating mtDNA in plasma exists in both particle-associated and non-particle-associated forms. The increase in levels of circulating mtDNA seems not to discriminate between benign and malignant lesions [18], [20]. However, this simple blood test provides a prognostic biomarker, which (in a combination with other disease-specific tests) can i.e., predict biochemical tumor recurrence after tumorectomy [21].

We have wondered, whether the quantification of mtDNA levels also allows to detect vulnerable risk groups after exposure to toxic-carcinogenic chemicals? The current study investigates this aspect using serum samples from individuals exposed to halogenated fumigants and shows the diagnostic relevance of circulating mtDNA measurements in the blood of the present cohort.

## Materials and Methods

### Study Subjects

We recruited 164 subjects with presumed occupational or environmental intoxication to fumigants. Subset of individuals (n = 78, 47%) with no indication for the exposure to halogenated hydrocarbon fumigants were excluded from the current analysis, because the exposure to halo-methane or halo-ethane could not be confirmed. Matched serum and urine samples of 86 subjects (78% male and 22% female subjects at median age of 44; 66% smoker and 34% non-smoker) exposed to halogenated hydrocarbon pesticides (methyl bromide, ethylene dichloride, dichloromethane) and 30 non-exposed control persons (53% males and 47% females at median age of 40; 33% smoker and 67% non-smoker) were further analyzed. The patients and non-exposed controls were diagnosed between 2006 and 2012. All had given written informed consent and were asked about possible contacts to pesticides or solvents in the past. None of the patients or controls had renal or hepatic disorder at the time of examination as assessed by the clinical history and Combu-urine-tests (Hoffmann-La Roche, Switzerland). The median body mass index (BMI) was with 27 slightly higher for the patient group than with 24 for the control subjects. All participants were Caucasian. If medically indicated, clinical feedback was provided to participants. As a part of the study design, no other data including risk status were provided to the participants. Patients who met the diagnostic criteria for exposure were further grouped.

### Personal Exposure Assessment

All 194 subjects (164 patients and 30 controls) filled in a questionnaire concerning the occupational and environmental exposure to halo-methans, halo-ethanes, solvents, possible alcohol abuse and smoking status (questionnaire as supplementary material, S1). Occupational expert anamnesis was accomplished with additional ambient monitoring (if possible), and onside air measurements in warehouses or contaminated products off-gassing analysis (taken from the place of exposure). The medical records revealed the patient group with neurological and/or respiratory symptoms typical for exposure to fumigants as described previously [22]. Participants underwent clinical assessment of fumigant intoxication which ascertained occupational and medical histories as well as physical examination including a neurologic evaluation and lung function testing.

The analyses of air and body fluids (blood, urine) samples included screening (SCAN) and targeted (SIM) analysis of halogenated hydrocarbons, plus possible co-exposure to solvents, such as benzene, toluene, styrol, xylol or ethyl benzene performed by gas chromatography-mass spectrometry (GCMS). Air analysis methods were described elsewhere [23], [24]. The human biomonitoring analysis included GCMS (head space) analyses of methyl bromide (m/z 94,96,79), ethylene dichloride (m/z 62,49,96), methyl dichloride (m/z 84,49,86) and other halo-methans/−ethans in blood (using head-space method) based on validated methods from the study group analysis of biological material by the senate commission of the German Research Council, DFG [25]. Bromide was detected using photometric method as described previously [26]. Bromide ion released from methyl bromide as a result of the reaction with L-glutathione and alkylation of macromolecules can be measured with a half-life of 16 days and a cut-off value of 5 mg/L serum. Long term biological exposure effects were determined by measuring reaction products (adducts) with macromolecules in blood, such as the hemoglobin adducts methyl valine and hydoxy ethyl valine. To determine adducts of N-terminal valine in hemoglobin, the erythrocytes were extracted from whole blood, and the cells were destroyed through lysis. The globin was precipitated out of the hemoglobin solution. The internal standard N-2-ethoxyethylvaline-alanine-anilide was added. The alkylated N-terminal valines (N-2-cyanoethylvaline (CEV), N-2-hydroxyethylvaline (HEV) and N-methyl valine (MEV)) were converted to their derivate using penta-fluoro-phenyl-isothiocyanate and cleaved from protein by means of modified Edman degradation. Globin fractions were separated by capillary gas chromatography. The quantitative determination was carried out by mass spectrometry (m/z = 377 and m/z = 335 for N-2-cyanoethylvaline, m/z = 308 and m/z = 350 for N-2-hydroxyethylvaline, and m/z = 338 and m/z = 310 for N-methyl valine). We could determine the hemoglobin adducts with 80% sensitivity and 65% specificity. Current smoking status was objectivized by nicotine/cotinine biomonitoring using the GCMS method [27] for the short term exposure (hours, days). The long term exposure (months) was evaluated using the analysis of cyano-ethyl-valine adducts (see above for details of the method) and the past exposure was evaluated by a questionnaire (ex-status). Creatinine was determined using the HPLC method as described previously [27]. 8-OHdG was determined with enzyme linked immunoabsorbent assay provided by JaICA’s (New 8-OHdG check-kit, www.jaica.com). All analytical methods were validated using inter and intra laboratory quality assessments (www. g-equas.de, external quality assessment from the German Society for Occupational and Environmental Medicine).

### Analysis of Circulating DNA in Blood and Urine

For the extraction of circulating DNA from serum obtained after centrifugation of blood samples in S-Monovette tubes (Sarstedt, Germany) and urine samples, DNA was prepared using the semi-automatic Qiacube. The same preparation method was used for all patients and control samples (using either serum or urine specific kits). The QIAamp Circulating Nucleic Acid Kit (Qiagen, Hilden, Germany) was used to isolate circulating DNA from 1 mL of serum and 4 mL of urine. Circulating DNA was isolated according to the manufacture’s protocol and spectrophotometrically (Nano Drop 2000, Thermo Fischer, USA) quantified. Quantitative analysis of mtDNA in serum and urine was performed by quantitative real-time PCR (qPCR) [28]. We used two primer sets specific for the mitochondrial ribosomal 16S-RNA: The first primer pair amplified a 79-bp fragment (mtDNA-79) that represents total mtDNA and includes DNA released by apoptotic cells. The second primer pair amplified a 230-bp fragment (mtDNA-230) that corresponds to mtDNA released by non-apoptotic types of cell death (i.e. necrosis) or active secretion. The sequence of the forward primer specific for both mtDNA fragments was 5′-CAGCCGCTATTAAAGGTTCG-3′. The sequence of the reverse primer specific for mtDNA-79 was 5′-CCTGGATTACTCCGGTCTGA-3′, of the reverse primer specific for mtDNA-230 was 5′-GGGCTCTGCCATCTTAACAA-3′. To date, no mutations have been reported in these amplified regions. The degree of fragmentation was expressed by the mtDNA integrity and defined by the ratio of the relative amounts of mtDNA-230 to mtDNA-79. The qPCR was performed in triplicate on an ABIPrism 7900 HT (Applied Biosystems, USA). Each 10 µL-reaction consisted of 1 µL DNA, 5 µl SYBRGreen (Invitrogen, Scotland) and 0.25 µL forward/reverse primer. Each run included water blanks as a negative control. PCR conditions were 95°C for 15 min, followed by 40 cycles at 95°C for 15 s, 60°C for 30 s and 72°C for 30 s. For melting curve analysis (to confirm specificity of the PCR products) an additional cycle was added with 95°C for 15 s, 60°C for 15 s and 95°C for 15 s at the end.

### Data Analysis

The data derived from the qPCR were calculated and evaluated by the ΔCt method as follows: ΔCt = mean value Ct (reference GAPDH) - mean value Ct (79- or 230-bp DNA fragments). The relative DNA levels corresponded to the 2∧(ΔCt) values. These data were statistically evaluated using both the Statistical Software Package SPSS v. 20.0 and GraphPADPrism4 (Motulski 1999). For non-parametric comparisons of multiple independent variables the Kruskal-Wallis test (ANOVA) was used. To differentiate between exposed and non-exposed populations the Dunn’s multiple comparison test of a 95% CI (confidence interval), Bonfferoni and multiple linear regression analyses were performed using GraphPad Prism V4/V6 [29]. For the meta-analysis Fisher’s combined probability test was applied. To estimate the sensitivity and specificity of the analysis, receiver operator characteristic analysis (ROC) was carried out. For non-parametric comparisons, univariate analyses of the Mann Whitney-U test of two independent variables were used. Areas under the curves (AUC) were calculated, assuming nonparametric distribution. P values from <0.05 was considered as significant.

### Ethics Statement

The study was approved by the local ethic commission (Ethik-Kommission der Ärztekammer Hamburg, No: PV3597 to XB), based on the German State and Federal Law, in accordance with the European ICH-GCP, Good Clinical Practice Guideline Rules.

## Results

### Exposure Assessment Analysis

**Figure 1:**
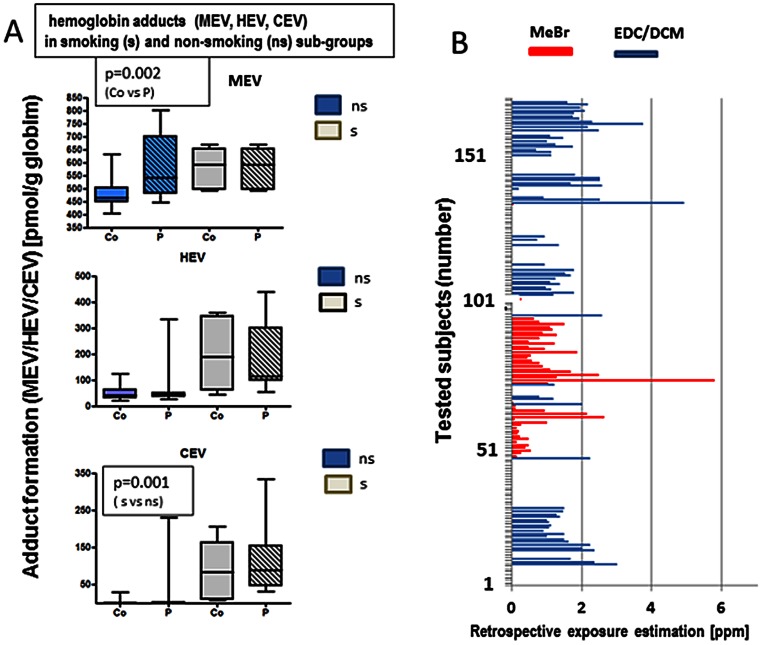
Exposure assessment in subjects with presumed intoxication with halo-alkane-based pesticides (fumigants). A. Formation of hemoglobin adducts: MEV (upper panel), HEV (middle panel), CEV (lower panel) in smoking (s) and non-smoking subgroups (ns). The data are shown as box plots indicating the minimum, the 25-percentile, the 75-percentile values; the medians are shown as a cross line. Blue boxes show non-smoking subgroups (ns) while the grey ones represent smoking subgroups (s); for both subgroups (s, ns) the empty boxes show data for the non-exposed controls (Co), the boxes with the dashed lines show exposed individuals (P). B. Retrospective exposure estimated for all tested subjects with presumed intoxication to methyl- or ethyl halide pesticides. As described in the methods, the exposure was weighted from the human biomonitoring data and expert valuation of the job history and clinical data. The lines show calculated exposure values to methyl bromide (red) or ethylene dichloride/dichloromethane (blue). The concentrations are shown in ppm (mL/m^3^).

The exposure assessment was performed using different approaches, including human biomonitoring (methyl bromide, ethylene dichloride, dichloromethane, screening for other halogenated hydrocarbon fumigants, solvents), a standardized, structured, self-administrated questionnaire (as supplementary material, S1), medical records and the comprehensive expert analysis (including the occupational exposure history and full clinical diagnosis of intoxication symptoms). As a part of the exposure assessment, we measured formation of the hemoglobin adducts methyl valine (MEV) and hydroxyl-ethyl-valine (HEV) in all patients. (Fig. 1 A). These data were included in the individual exposure evaluation together with the evaluation of individual clinical and environmental co-exposure data considering personal life style risk factors (i.e., smoking status). In the non-smoking subgroup there was a significant difference of the methyl valine (MEV) adduct formation between exposed groups and controls (P = 0.002). In the smoking subgroup the evaluation of individual smoking status was adjusted. Since smoking is a confounder for the formation of hydroxyl-ethyl-valine adducts, the HEV data derived from smoking individuals were not considered for the exposure evaluation. Interestingly, we could observe a gender-based difference. Women harbored higher HEV values than men (P = 0.007). The formation of cyano-ethyl-valine adducts (CEV) indicating the arylnitril exposure found in cigarette smoke showed no difference between exposed (P) and control (Co) subjects. However, as expected, there was a significant difference between smoker and non-smoker (P = 0.001). In the current study CEV was used as a long-term life-style biomarker (for smoking status).

All exposure assessment data were used to evaluate exposure of individual subjects. Based on the expert judgment classifying various dimensions of exposure (job history, probability, intensity, frequency of exposure), the biomonitoring data and the medical records (clinical intoxication symptoms), we have excluded 47.5% of the subjects in which the exposure to halo-methans or halo-ethans could not be confirmed. In this regard, the collected data allowed us to calculate the extrapolated exposure values (Fig. 1B). To note, only exposure to methyl bromide and ethylene dichloride/dichloromethane were calculated. The low concentrations of other halo alkanes could be neglected for the analyzed group. Ethylene dichloride and dichloromethane were grouped together, because the majority of subjects had cumulative exposure to both agents. Figure 1B shows these correlated exposure equivalent values for individual subjects with exposure to methyl bromide as red lines and to both other halogenated fumigants (ethylene dichloride and dichloromethane) as blue lines.

With respect to exposure to hydrogenated hydrocarbon pesticides, three groups of patients with confirmed exposure (n = 86) were assembled. Patient subgroup P1 (n = 18) with confirmed current intoxication (i.e., parent substances still present in blood), P2 (n = 20) with short-term past exposure (weeks, up to 4 months) as evaluated by increased metabolites/adducts found in concentrations above the reference cut-off values, and P3 (n = 48) with long-term past exposure based on case history and clinical symptoms (with no current exposure, i.e., neither halogenated hydrocarbon fumigants nor their metabolites in blood or urine, interim time at least 6 months). The initial incorporated exposure values were in the range between 0.100 and 5.8 ppm (extrapolated, predicted values) based on exposure correlation from human biomonitoring data (P1, P2) and expert judgment (P3).

### Increased Total DNA Levels in the Serum of Exposed Patients

**Figure 2:**
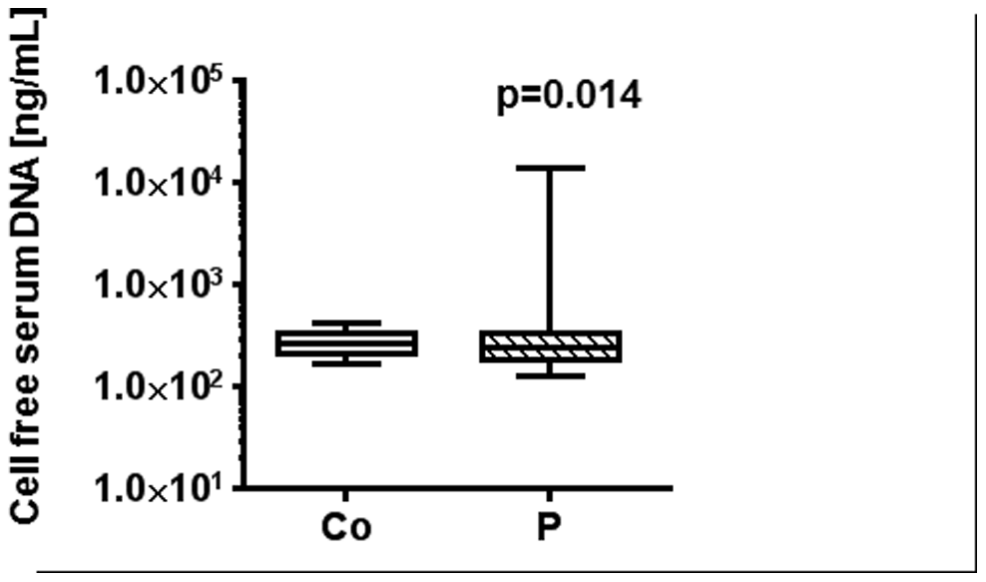
Increased levels of circulating DNA after exposure to halo alkanes. The box plot (the median values are shown as a cross lines) implicates total cell-free serum DNA levels in patients exposed to halo-methane/or -ethane fumigants (exposed subjects, P) as compared with the healthy control group (Co). The significance was determined by Kruscal-Wallis test and Dunn’s post-test (P = 0.014).

The comparison of the serum levels of cell-free DNA showed a slight, but significant increase in exposed patients vs. controls (p = 0.014). The median serum DNA levels were approximately 1.8-fold higher in exposed patients (n = 86) than in healthy individuals (n = 30) (Fig. 2). The normality tests (Gaussian distribution) revealed for the serum levels of total DNA: p<0.001 for P1, p = 0.531 for P2, p = 0.001 for P3. To explore the potential use of DNA from urine as a source for circulating DNA, we quantified total DNA levels in the urine samples of exposed patients and healthy individuals. However, we did not detect different DNA levels in both cohorts suggesting that urine is not suited for the quantification of cell-free DNA (data not shown).

### Changes of mtDNA Levels and mtDNA Integrity in the Serum of Exposed Patients

Furthermore, we quantified the relative serum amounts of mtDNA fragments (mtDNA-79 and mtDNA-230) which are different in size and assumed to be derived from different cell deaths. DNA from cells undergoing apoptosis is cleaved to inter-nucleosomal fragments of usually 185 to 200 bp in length. This uniformly truncated DNA is produced by a programmed enzymatic cleavage process during apoptosis. DNA from necrotic cells is distinctly larger (>10 kbp). The mtDNA-79 primer set used in our qPCR amplifies a 79-bp DNA fragment, and thus, allowed quantification of total mtDNA levels independent of the different DNA lengths and cell-deaths. The mtDNA-230 primer set amplifies a 230-bp (>200-bp) DNA fragment of non-apoptotic origin.

**Figure 3:**
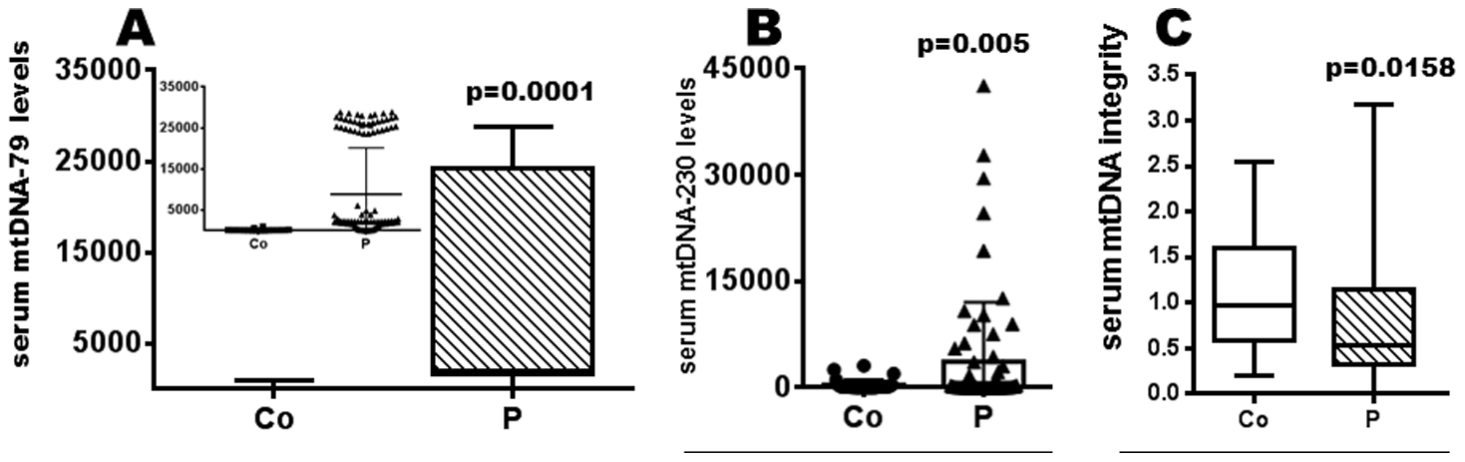
Exposure to halo alkanes enhances relative serum levels of mtDNA. Relative serum levels of mtDNA-79 (A), mtDNA-230 (B) or the mtDNA integrity in controls (Co) and exposed (P) groups. A. The distribution of circulating mtDNA-79 is presented as a box plot indicating all data from minimum to maximum and median values (as cross line). The inset additionally shows a jittered scatter plot and its mean ±SD values. The additionally performed meta-analysis (Fisher’s method) revealed a p-value of 0.0042. B. The distribution of circulating mtDNA-230 shown as a scattered plot (min./max.) with incorporated box plot. C. A whisker box plot comparing the mtDNA integrity between the exposed patients (P) and healthy unexposed individual is shown.

Our analyses showed that the median serum levels of mtDNA-79 (p = 0.0001, Fig. 3A) and mtDNA-230 (p = 0.005, Fig. 3B) were significantly higher in exposed than non-exposed individuals. The meta-analysis revealed a p value of 0.0042 (serum levels of mtDNA-79 for exposed individuals, n = 86, versus controls, n = 30). The statistical evaluation showed that there was no correlation between the relative mtDNA-79 and mtDNA-230 levels indicating the different release mechanisms (data not shown). The normality tests (Gaussian distribution) revealed for the serum levels of mtDNA-79 in the individual patient subgroups: p<0.001 for P1, p<0.001 for P2, p<0.001 for P3. In addition, we quantified the levels of mtDNA-79 and mtDNA-230 in the urine samples of both cohorts. These analyses showed no difference of the mtDNA levels between these cohorts (data not shown).

Finally, we examined the mtDNA integrity which was defined as ratio of mtDNA-230 to mtDNA-79 fragments. The DNA integrity (quality) measured in the blood may represent cell death and characterizes the DNA fragmentation pattern. The DNA integrity index is, thus, represented by the ratio of longer to shorter DNA fragments (ratio of mtDNA-230 to mtDNA-79). Unlike absolute DNA levels, which do not reflect how the DNA is released, DNA integrity specifically represents the relative amount of non-apoptotic cell deaths. The clearance rate of DNA in the blood of the patients can directly contribute to the blood DNA level. In contrast, it does not significantly influence the values of DNA integrity because both the amounts of longer and shorter DNA fragments may be similarly affected.

Our analyses show that in exposed patients the mean DNA integrity was significantly different from the integrity of healthy individuals (Fig. 3C, p = 0.0158). The mtDNA integrity is decreased and the portion of short DNA fragments (mtDNA-79) is increased in exposed patients reflecting increased apoptotic processes.

**Figure 4:**
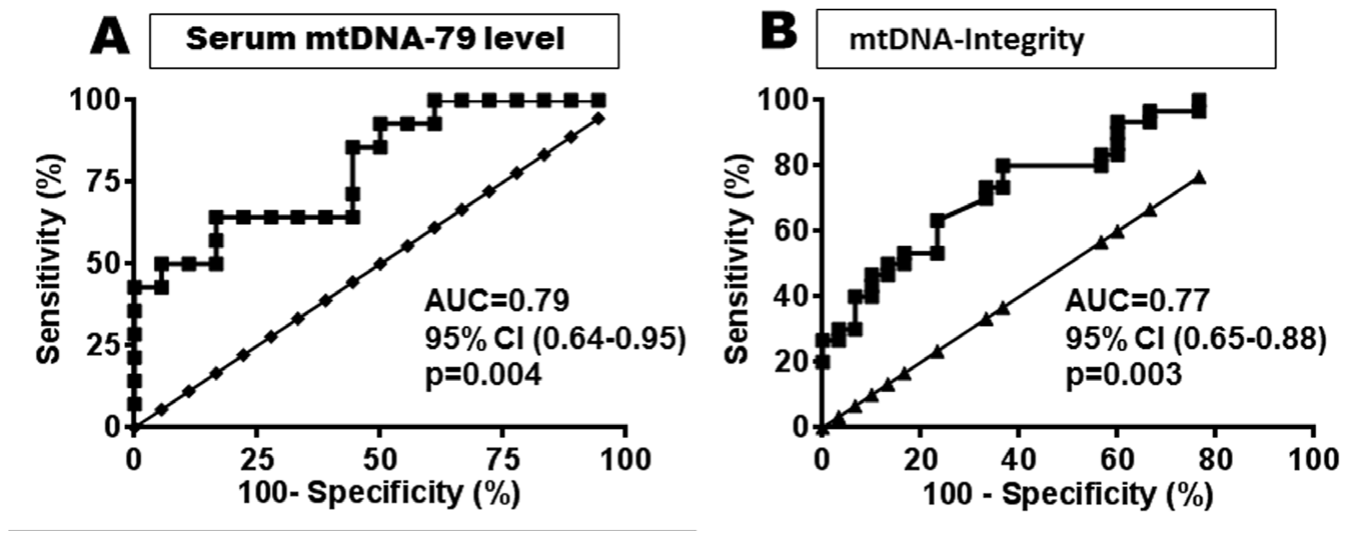
mtDNA-79 fragment has a potential to serve as a sensitive biomarker recognizing vulnerable groups after exposure to halo alkanes. Receiver operating characteristic (ROC) curves of cell-free serum mtDNA-79 levels (A) and mtDNA integrity (B) discriminate between patients exposed to methyl/ethyl halides and healthy controls. Sensitivity of the individual biomarkers is plotted on the y-axis, whereas 1-specificity or the false positive rates plot on the x-axis. The data are shown with 95% CI. The mtDNA-230 data are not shown (AUC = 0.5, p = 81). AUC, area-under-curve; CI, confidence interval.

The significant discrimination between exposed patients and healthy individuals by the different levels of circulating mtDNA-79 and mtDNA-230 as well as the DNA integrity was also documented by their AUC values of 0.790 (Fig. 4A), 0.515 (data not shown) and 0.770 (Fig. 4B), respectively. The analysis of the sensitivity versus specificity for the serum mtDNA-79 levels were 79% versus 65% (a cut-off value of >122), and for the mtDNA integrity the assay sensitivity was 25% and the specificity 77% (a cut-off value of 0.085).

The parametric ROC curve analysis, which correlated true- and false-positive rates, suggests using 95% confidence interval that the serum level of mtDNA-79, but not mtDNA-230, may serve as a useful effect biomarker to discriminate between the subjects exposed to halogenated hydrocarbon fumigants and the control cohort.

### Correlations with the Data of Exposure

**Figure 5:**
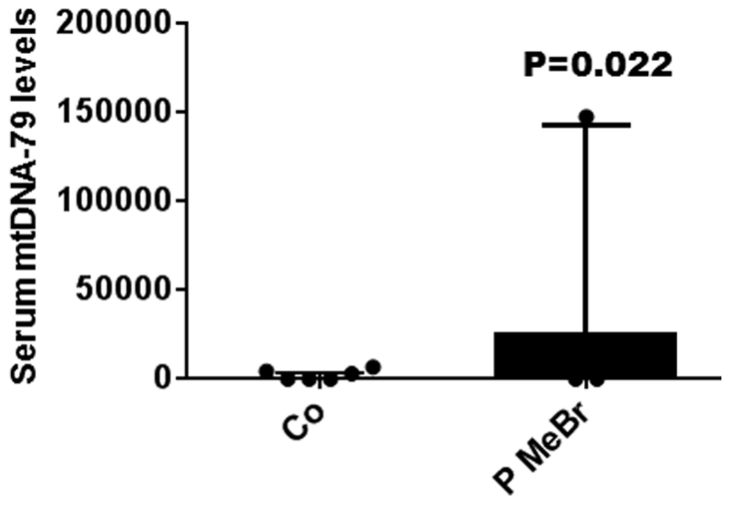
Exposure to methyl bromide causes a slight increase in serum mtDNA. The box plot (the median values are shown as cross lines) implicates the relative mtDNA-79 levels in serum of patients exposed to methyl bromide (MeBr, P) as compared with the healthy control group (Co).

**Figure 6:**
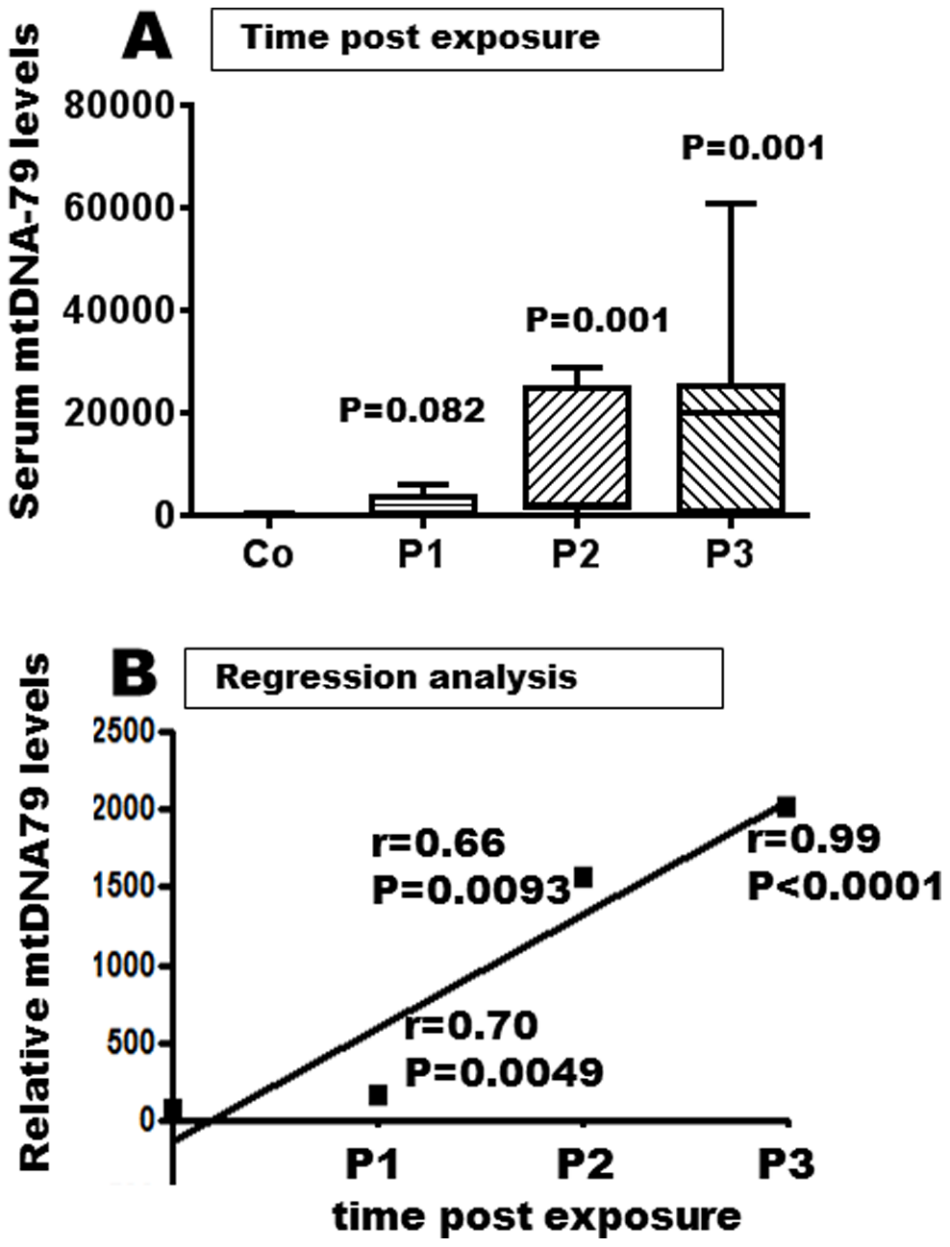
Time related augmentation of serum mtDNA after exposure to haloalkanes. A. The box plot (the median values are shown as cross lines) implicate serum levels of cell-free mtDNA-79 in patients exposed to halo-alkanes. The exposed subjects are grouped in P1, P2 and P3 subgroups according to the time post-exposure as compared with the healthy control group (Co). B. The regression analysis shows the rise in the relative amounts of serum mtDNA after exposure to halo-alkanes as a relation to the time post-exposure.

We did not find any correlations of the levels of circulating mtDNA and mtDNA integrity with either the chemical concentrations or confounder levels (data not shown). However, the increased levels of mtDNA-79 were associated with patients exposed to methyl bromide (p = 0.0022, Fig. 5). The various extension of co-exposure with ethylene dichloride and dichloromethane did not allow us to discriminate further exposure groups. Considering the three patient subgroups (Fig. 6A), a clear trend of increasing levels of mtDNA-79 was associated with the lag time post-exposure. Hence, there was a continuous rise of the levels of mtDNA-79 in the serum of P1 (n = 18) with confirmed current intoxication (p = 0.0082), over P2 (n = 20) with short-term (20–120 days) past exposure (p = 0.0001) to P3 (n = 47) with long-term (>5 months) past exposure (p = 0.0001). The multiple linear regression analysis emphasizes this significant time related trend (Fig. 6B). For the levels of mtDNA-230 and mtDNA integrity, we could not observe such a relationship to the exposure to methyl bromide or other ethylene dichloride/dichloromethane (data not shown).

We looked closer at the individual patient groups and further performed ROC analysis. The areas under curve values (AUC) values of serum mtDNA-79, mtDNA-230 and mtDNA integrity for the three patient subgroups were separately compared (with values of 0.669, 0.620 and 0.724 for the P1, P2 and P3 subgroups, respectively). In addition, specificity and sensitivity were calculated for the serum mtDNA-79. The data reveal that the assay is more sensitive for the subjects with a shorter post-exposure time with 91% for P1 (a cut-off of 39), 67% for P2 (a cut-off of 173) and 33% for P3 (a cut-off of 7.2), whereas its specificity increases with time post-exposure (with 30% for P1, 70% for P2 and 97% for P3).

### No Discrimination between the Patient and Control Cohorts by 8-OHdG

These findings motivated us to evaluate if a marker of oxidative DNA damage is also parallel elevated in the exposed group. Hydroxylation at the C8 position of guanine is known to cause DNA strand breaks and oxidative damage of DNA. One of the most abounded oxidative DNA bases is 8-hydroxy-2′-deoxyguanosine (8-OHdG) used as a biomarker. 8-OHdG is released into the circulation after genomic DNA repair by the DNA base excision pathway and eliminated into urine [30], [31]. We have measured 8-OHdG in the urine samples and could not detect any increases in the levels of 8-OHdG among the groups, suggesting no correlation between the increased levels of circulating mtDNA and 8-OHdG release (data not shown). When we examined the patient individual data slightly increased, but not significant values were observed within the subgroups P1 and P2, which, however, did not warrant further analyses. On the other hand, we observed a marginally significant decrease in the amounts of secreted 8-OHdG in the patient subgroup P3 compared with the control cohort (p = 0.037).

## Discussion

This is the first study investigating the levels of total and mtDNA in the blood circulation of patients exposed to halogenated hydrocarbon pesticides (methyl- and ethyl halides fumigants). We detected that the concentrations of total DNA, mtDNA-79 and mtDNA-230 are increased, and the mtDNA integrity is decreased in the serum of exposed patients compared with those in healthy individuals. The increasing levels of mtDNA-79 were associated with the increase in the lag time post-exposure to halo alkanes. There was a continuous rise of the levels of mtDNA-79 in the serum of patient subgroup P1 with confirmed current intoxication (enhanced exposure biomarker in human biomonitoring), over P2 with short term past exposure (presence of metabolites or adducts) to P3 with clear clinical intoxication symptoms and longest interim time/term past exposure. Surprisingly, the urine values of 8-OHdG did not discriminate exposed patients from healthy individuals, and consequently, were not associated with the serum levels of total DNA and mtDNA. Thus, quantification of circulating cell-free serum DNA, in particular mtDNA, could become a minimally invasive tool for screening exposed patients and an effect biomarker for post-exposure to methyl bromide or other methyl halides. In contrast, the quantification of total DNA and mtDNA in the urine samples did not differ exposed patients from healthy individuals. This could be explained by inhibitory factors, e.g., degrading enzymes, in the urine that may be more limiting than in serum. Conversely, other studies showed that circulating mtDNA may be increased in the urine in diseased individuals.

Halogenated hydrocarbon pesticides, such as methyl bromide, do not persist in the body for a long time, so that the exposure biomonitoring even after a serious intoxication is only possible for up to c. 3 weeks after exposure. Afterwards an additional effect monitoring (hemoglobin adducts) is possible for further 4 months. In any time an expert exposure evaluation is needed. Based on our experience, we weighted the exposure for each patient. The estimated exposure concentrations were in the range between 0.100 and 5.8 ppm. It is of note that the levels of halogenated hydrocarbon pesticides were lower in the majority of subjects than the occupational exposure limit of 1 ppm for methyl bromide or ethylene dichloride. However, the estimated exposure level was higher for all exposed patient subgroups than the reference doses drawn for toxic environmental effects of halogenated hydrocarbon fumigants. The California Office of Environmental Health has settled the non-cancer reference dose (RfD) value at 0.05 ppm (neurologic targeted toxicity) for acute air exposure to methyl bromide and at 0.005 mg/m^3^ (0.0012 ppm) for the respiratory tract target of chronic RfD (based on degenerative and proliferative lesions of the olfactory epithelium of the nasal cavity) (see [6], [23] for the reference values). The data in the literature on environmental seasonal exposure to halogenated hydrocarbon fumigant and methyl bromide show increased cancer incidences [2], [33] after exposure as low as ∼0.005 ppm. Additionally, the co-exposure to other CYP substrates was separately evaluated and weighted. Solvents (or alcohol) are known to lower CYP2E1 (in case of dichloromethane and methyl bromide) which results in higher GST-T1 metabolism and increased toxicity and risks [34], [35] .

In our study, we found that mtDNA-79 was most increased in the serum of P3 subgroup with clinical intoxication symptoms suggesting that these lesions lead to a long term release of mtDNA79 into the blood circulation. Xenobiotic halo-alkanes (halo-methanes or halo-ethanes) used in many industrial processes or as a part of various fumigant formulations (applied in global transport and storage) can elicit selective toxicity and may have carcinogenic potential. The intoxication targets after exposure to these methyl or ethyl halides (such as methyl bromide, dichloromethane, iodine methane, ethylene dichloride, propylene dichloride or tetra chloromethane) may involve many organs, such as liver, lung or various brain regions, causing, i.e., neuronal cell death (both apoptotic and necrotic) in the cerebellum, while sparing granule cells of hippocampus [36]. Some identified detoxification pathways involve CYP oxidation and formation of glutathione (GSH)-S-conjugates either in the periphery or in the brain, which are metabolized and excreted into the urine as mercapturic acid. The mechanisms of neuronal cell death with methyl halides involve DNA damage, methylation and inhibition of DNA repair mechanisms plus depletion of the intracellular antioxidant GSH and oxidative stress [36]. Only little is known on the potential molecular mechanisms of intoxication and cell death by ethyl halides. Both genomic and mitochondrial DNA fragmentations are likely.

To date, several benign diseases and different cancer entities have been linked with increased levels of circulating, cell-free DNA. The reasons for these high levels in the patient serum are unclear, but could be explained among others by the fact that benign and malignant, as well as exposure-based lesions reflect similar pathological and inflammatory processes. In our current study, the highest DNA levels were the levels of mtDNA-79 detected in the serum of patients with long-term past exposure. The considerable DNA discharge into the blood circulation of this patient subgroup could imply the high activation potential of inflammatory processes after long-term past exposure. Inflammatory processes are usually accompanied by an increased apoptotic cell death confirming our observation of the decreased mtDNA integrity in the serum of exposed patients. The DNA integrity which reflects the DNA fragmentation pattern in the blood revealed a higher portion of short DNA fragments in exposed patients. Since necrotic cells release long DNA fragments, these short DNA fragments may represent total DNA including fragmented necrotic and apoptotic DNA. How long it will take until these DNA levels normalize, and whether the extent of exposure can influence the time to normalization is not known. However, these persistently increased levels of cell-free DNA can be assumed to represent an increased disease risk or persistence.

Carcinogenic effects of methyl- and ethyl halides have been associated with both genotoxic and non-genotoxic mechanisms, reflecting e.g., the activation of cellular oxidation and stress [6], [8], [37], [38]. It is likely that elevated mtDNA levels reflect early precancerous lesions at a stage that presumably still permits active repair mechanisms. Mitochondria underlying transitions form primary clinical lesions in association with the environmental exposure [39]. These changes include enhanced oxidative stress and mitochondrial dysfunction. Somatic mtDNA has been increasingly observed in primary human cancers [40], [41]. It has been suggested that the nucleus attempts to compensate for energy deficiency in cells harboring deleterious mtDNA by producing more mitochondria. The mitochondrial energetic output declines, the production of reactive oxygen species (ROS), which act as both DNA mutagen and cellular mitogen, increases, and the propensity of apoptosis advances [42]. In this regard, the role of mitochondrial energy imbalance in the solid tumor etiology is well documented [39]. Surprisingly, we could not observe any increase in ROS production using urinary 8-OHdG as a marker. However, the molecular mechanism underlying the cellular ROS and energy imbalance is far more complex, and encompasses several pathways and enzymes, i.e., superoxide dismutase, heme-oxigenase, along with CYP oxidases and glutathione transferases [43], [44], which we did not investigate in our study. A further important aspect is that exposure to haloalkane fumigants, like many other environmental chemicals, can induce both degenerative diseases and cancer, both are complex multistep diseases which may share some common pathways including changes in ROS and apoptosis [45]. Depletion of glutathione plays a mechanistic role in halogenated hydrocarbon-induced neuronal toxicity [36] and also in mitochondrial permeability transition [46]. The increased prevalence of mtDNA-79 containing apoptotic and necrotic DNA fragments and the decreased DNA integrity in our exposed patient cohort allows us to stipulate the glutathione-driven process as a possible mechanism.

Considerable attention have recently been focused on pathobiology of mitochondrial (energetic) driven diseases, and numerous health problems have been suggested to be etiology associated with environmental exposure [42]. An increase in the mitochondrial DNA level has been documented after environmental and occupational exposure to benzene highlighting the role of mtDNA in exposure associated with intoxication and cancer [47], [48]. Carugno et al. [47] could clearly correlate an increase in mtDNA copies in blood cells and plasma after exposure to low (<100 ppb) benzene concentrations (along with epigenetic changes on nuclear DNA). Our findings corroborate and extend those data and show that high mtDNA levels are associated not only with exposure to carcinogenic benzene, but also with halo alkanes (halo methanes/ethanes). However, the data also address many questions regarding the involvement of possible pathways and repair mechanisms. Further studies are needed to elucidate the mechanisms underlying the release of DNA into the blood circulation. The limitation of our study is the small patient cohort. On the other hand, we could rely on both objective personal exposure assessment and evaluation of confounder levels (including personal biomarker level, ambient monitoring data, comprehensive expert judgment, environmental/occupational history analysis and clinical examination for intoxication symptoms).

### Conclusions

The major question whether circulating mtDNA is a valuable risk parameter can only be answered in large scale follow up cohort studies. We suggest circulating mtDNA as biomarker reflecting mitochondrial destabilization in the target organ after environmental exposure to toxic carcinogenic chemicals. The advantage of using circulating mtDNA is that it delivers information direct from affected organs/cells without a detour to surrogate tissue. An additional advantage of this marker is that it provides an option for a retrospective analysis of serum samples and warrants for further (follow up) studies, especially to identify possible genetic alterations.

## Supporting Information

File S1 **Fumigant Exposure Questionnaire: FumEx1.** Self administrated questionnaire for patients with presumed exposure to fumigants.
(PDF)
