# Supplementary material for: Circulating Mitochondrial DNA as Biomarker Linking Environmental Chemical Exposure to Early Preclinical Lesions Elevation of mtDNA in Human Serum after Exposure to Carcinogenic Halo-Alkane-Based Pesticides
Source: PLoS One. 2013 May 31;8(5):e64413. doi: 10.1371/journal.pone.0064413 (PMC3669318; doi:10.1371/journal.pone.0064413)
Supplement: File S1 — Fumigant Exposure Questionnaire: FumEx1. Self administrated questionnaire for patients with presumed exposure to fumigants. (PDF) [file pone.0064413.s001.pdf]

FUMIGANT EXPOSURE QUESTIONNAIRE

Fum-Ex1

STUDY SITE (INSTITUTION) ____

CO-INVESTIGATOR ____

DATE OF EVALUATION ___/___/______

NAME-ACRONYM/or Nr. ____

DATE OF BIRTH ___/___/______ (MONTH/DAY/YEAR) or AGE ___ YEARS

SEX F___ M ___

HEIGHT (cm) _______

WEIGHT (kg) _______

CURRENT SMOKER: YES ___ NO ___ NEVER SMOKED ___

WHEN DID YOU START SMOKING? DATE: ___/___/______ (MONTH/ YEAR)

EX-SMOKER: YES ___ NO ___

DATE QUIT: ___/___/______ (MONTH/ YEAR)

SMOKING HISTORY # CIGARETTS ___ per DAY

OCCUPATIONAL HISTORY

1. JOB DESCRIPTION ____
2. SINCE WHEN ARE YOU WORKING IN YOUR CURRENT JOB?
   ___ (M)/ ______ (Y)
3. DO YOU HAVE CURRENTLY CONTACT WITH FUMIGANTS, PESTICIDES OR OTHER TOXIC CHEMICALS ?
   YES ___ NO ___
4. REGULARY? YES ___ NO ___
5. IF YES TO QUESTION # 3, SPECIFY: ___ Methyl bromide (Bromomethane)

   ___ Ethylene dichloride (1,2 Dichloroethane)

   ___ Methylene chloride (Dichloroethane)

   ___ Phosphine

   ___ ______________________________ (OTHER)

   ___ ______________________________ (SOLVENTS)

I. DURATION OF EXPOSURE IN TOTAL ______ (MONTHS)
II. HOW MANY HOURS DO YOU HAVE CONTACT WITH THE AGENTS MENTIONED ABOVE PER WEEK? ___ HOURS
III. WHEN WAS THE LAST EXPOSURE? ___/___/______ (MONTH/DAY/YEAR)
IV. DURATION OF LAST EXPOSURE ____ (DAYS) ____ (HOURS) ____ (MINUTES)

6. IF NO (QUESTION # 3): DID YOU WORK WITH THESE AGENTS IN THE PAST?
   YES ___ NO ___
   WHICH AGENT? ____
   WHAT WAS YOUR JOB DESCRIPTION AT THAT TIME? _________________________
   EXPOSURE STARTED (DATE) ___/______ (MONTH/YEAR)
   EXPOSURE ENDED (DATE) ___/______ (MONTH/YEAR)
7. WHILE WORKING DID YOU USE ANY PROTECTION EQUIPMENT? YES ___ NO ___
   IF YES: WHICH? ____
8. SYMPTOMS
   HOW MANY TIMES DID THE FOLLOWING SYMPTOMS OCCUR DURING OR AFTER WORK IN THE LAST 12 MONTHS?

SYMPTOMS/INCIDENCE	ALMOST ALWAYS	OFTEN	SPORADIC	ALMOST NEVER	NEVER	WHEN DID IT OCCURE FOR THE FIRST TIME? (M/D/Y)
HEADACHE	I_I	I_I	I_I	I_I	I_I	___/___/______
DIZZINESS	I_I	I_I	I_I	I_I	I_I	___/___/______
AIRWAYS IRRITATION, COUGH	I_I	I_I	I_I	I_I	I_I	___/___/______
MUCOSA IRRITATIONS (EYE ITCHING, RHINITIS,STOMATITIS)	I_I	I_I	I_I	I_I	I_I	___/___/______
SKIN IRRITATION	I_I	I_I	I_I	I_I	I_I	___/___/______
NAUSEA	I_I	I_I	I_I	I_I	I_I	___/___/______
DIZZINESS	I_I	I_I	I_I	I_I	I_I	___/___/______
MUSCLE CRAMPS	I_I	I_I	I_I	I_I	I_I	___/___/______
CONCENTRATION DISORDERS	I_I	I_I	I_I	I_I	I_I	___/___/______
DYSGUSIA Distortion sense of taste	I_I	I_I	I_I	I_I	I_I	___/___/______
NUMBNESS	I_I	I_I	I_I	I_I	I_I	___/___/______
DIARRHEA, ABDOMINAL CRAMPS	I_I	I_I	I_I	I_I	I_I	___/___/______
WEAKNESS, FATIGUE	I_I	I_I	I_I	I_I	I_I	___/___/______
SEIZURES	I_I	I_I	I_I	I_I	I_I	___/___/______
DISTURBANCE OF MEMORY	I_I	I_I	I_I	I_I	I_I	___/___/______
CHEST TIGHTNESS, DYSPNEA	I_I	I_I	I_I	I_I	I_I	___/___/______
EMOTIONAL INSTABILITY	I_I	I_I	I_I	I_I	I_I	___/___/______
SLURRED SPEECH	I_I	I_I	I_I	I_I	I_I	___/___/______
SLEEP DISORDER	I_I	I_I	I_I	I_I	I_I	___/___/______
IMPAIRED BALANCE,	I_I	I_I	I_I	I_I	I_I	___/___/______

DISTURBED GAIT						
TREMOR	I_I	I_I	I_I	I_I	I_I	___/___/______

10. HAVE YOU EXPERIENCED INCREASED COUGH WHILE WORKING? YES ___ NO ___

11. HAVE YOU EXPERIENCED INCREASED AIRWAYS IRRITATIONS WHILE WORKING?
    YES ___ NO___
12. HAVE YOU EVER BEEN UNCONSCIOUS IN THE LAST YEARS? YES ___ NO ___
13. IF YES (QUESTION #10): DID IT HAPPEN AT YOUR WORKPLACE? YES ___ NO ___
14. PLEASE INDICATE BELOW WHICH CHRONIC OR ACUTE CONDITION(S) YOU HAVE:
    I_I ARTHRITIS, SPECIFY ____
    I_I RHEUMATIC DISEASE, SPECIFY ____
    I_I ASTHMA, SPECIFY ____
    I_I CANCER, SPECIFY ____
    I_I DIABETES, SPECIFY ____
    I_I KIDNEY DISEASE, SPECIFY ____
    I_I LIVER DISEASE, SPECIFY ____
    I_I OTHER CHRONIC CONDITION, SPECIFY __________________________________
    ____
15. ARE YOU CURRENTLY TAKING ANY MEDICATION? YES ___ NO ___
    IF YES, SPECIFY: ____

16. DID YOU HAD CONTACT TO GENOTOXIC AGENTS?

    YES ___ NO ___

    WHICH AGENT? ____
17. HAVE YOU BEEN EXPOSED TO IONIZING RADIATION FOR DIGNOSTIC PURPOSES?

    YES ___ NO ___

    HOW LONG?____

18. ADDITIONAL INFORMATION IF NEEDED: ______________________________________

    ____

    ____

THANK YOU VERY MUCH FOR YOUR COOPERATION!
